# A case report on intensive, robot-assisted rehabilitation program for brainstem radionecrosis

**DOI:** 10.1097/MD.0000000000019517

**Published:** 2020-03-06

**Authors:** Francesco Tartamella, Antonino Chillura, Maria Francesca Pisano, Adele Cacioppo, Simona Licari, Deborah Caradonna, Simona Portaro, Rocco Salvatore Calabrò, Placido Bramanti, Antonino Naro

**Affiliations:** IRCCS Centro Neurolesi “Bonino-Pulejo," Messina, Italy.

**Keywords:** brainstem radionecrosis, cognitive rehabilitation, robot-aided gait training, robot-aided upper limb training, virtual reality

## Abstract

**Introduction:**

Radiotherapy is a valid treatment option for nasopharyngeal carcinoma. However, complications can occur following irradiation of the closest anatomical structures, including brainstem radionecrosis (BRN). The rehabilitation is poorly described in patients with BRN, despite its usefulness in improving functional independence in patients with brain tumors. We aimed at testing the usefulness of intensive, robot-assisted neurorehabilitation program to improve functional independence in a 57-year-old male with BRN.

**Patient concerns:**

A 57-year-old male diagnosed with a nasopharyngeal carcinoma, received a radiation total dose of 72 Gy. Owing to the appearance of a severe symptomatology characterized by dysphagia, hearing loss, and left sided hemiparesis, the patient was hospitalized to be provided with intensive pharmacological and neurorehabilitation treatment.

**Diagnosis:**

Follow-up brain magnetic resonance imaging disclosed no residual cancer, but some brainstem lesions compatible with BRN areas were appreciable.

**Intervention:**

The patient underwent a 2-month conventional, respiratory, and speech therapy. Given that the patient only mildly improved, he was provided with intensive robot-aided upper limb and gait training and virtual reality-based cognitive rehabilitation for other 2 months.

**Outcomes:**

The patient reported a significant improvement in functional independence, spasticity, cognitive impairment degree, and balance.

**Conclusion:**

Our case suggests the usefulness of neurorobotic intensive rehabilitation in BRN to reduce functional disability. Future studies should investigate whether an earlier, even multidisciplinary rehabilitative treatment could lead to better functional outcome in patients with BRN.

## 1 Introduction

Radiotherapy is a valid treatment option for head-neck malignancies, including nasopharyngeal carcinoma.^[1]^ However, complications can occur following irradiation of the closest anatomical structures, including brainstem. About that, brainstem radionecrosis (BRN) is a late, rare, and severe complication of radiotherapy, which can cause severe neurocognitive and motor dysfunctions, including impaired mobility, limitations in activities of daily living, risk for complications of immobility, falls, pain, anxiety/depression, with a consequent loss of functional independence and worsened quality of life.^[2–4]^

Corticosteroids and surgery are the main approaches to limit the evolutivity of BRN, reduce intracranial hypertension, and preserve the neighboring healthy brain.^[5]^ The usefulness of rehabilitative approaches in BRN survivors is instead poorly addressed in the literature,^[6]^ even though intensive rehabilitation have been shown to be useful in improving cognitive-motor dysfunction, survival, and quality of life in malignant primary brain tumors.^[7,8]^

There is growing evidence on the usefulness of rehabilitation robotics (using devices with motorized orthoses and weight support systems, exercise scenarios, and control strategies) aimed at facilitating the recovery of impaired sensory, motor, and cognitive skills.^[9,10]^ However, whether robot-aided rehabilitation of patients with BRN may be helpful in minimizing loss of neurological function, improving functional outcome, and reducing disability burden should be demonstrated. Herein, we report the case of a patient with BRN who was managed with an intensive, robot-assisted neurorehabilitation program, reporting on its usefulness.

## 2 Case description

**Figure 1:**
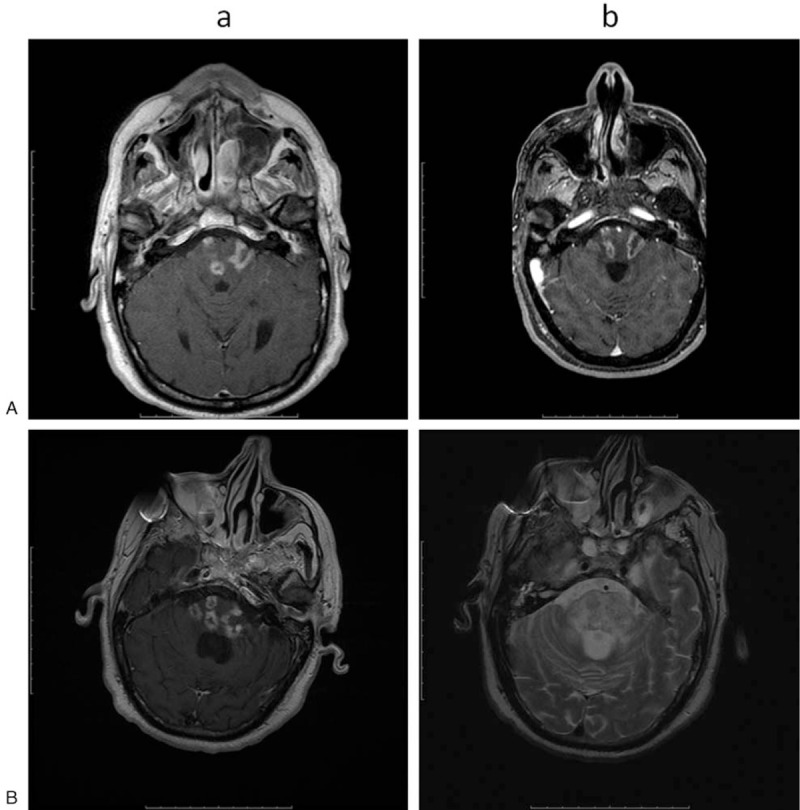
(A) Brain MRI at T10 showing multiple, nodular or curvilinear brainstem white matter lesions characterized by low signal in T1 (a) with heterogeneous contrast enhancement (“Swiss-cheese"), and high signal in T2 (edema and mass effect) (b). (B) Brain MRI at T11 showing more brainstem lesions with a worsening of the MRI features (edema and contrast enhancement). MRI = magnetic resonance imaging.

A 57-year-old male was diagnosed with a nasopharyngeal carcinoma (T4, N2, and M0) on January 2017. His past clinical history was unremarkable. He received a radiation total dose of 72 Gy in 2 Gy fraction, once a day, 5 days per week; an induction chemotherapy (2 cycles) using cisplatin was also provided. Brain magnetic resonance imaging (MRI) performed during the follow-up (T) at second (T2) and fourth (T4) month after radiotherapy showed no residual cancer. However, brain MRI at T6 showed some brainstem lesions characterized by T1-hypointensity, T2-hyperintensity, and heterogeneous contrast enhancement (Fig. 1A). These areas were hypometabolic at the Positron Emission Tomography. A diagnosis of BRN was thus made. The patient was still asymptomatic but he was provided with dexamethasone 8 mg bid. At T10, the patients was hospitalized as he began to complain a severe symptomatology characterized by left arm and facial paresthesia, dysphagia (as per 3-oz, Water Swallow Test), bilateral hearing loss, left facial nerve palsy, and left sided hemiparesis (as per Muscle Research Council scale) with unstable ambulation (as per Tinetti Performance Oriented Mobility Assessment); consequently, he showed a clear independence impairment (as per Functional Independence Measure) (Table 1). Systemic steroid therapy was continued. An intensive neurorehabilitation program was also prescribed. The rehabilitation protocol consisted of a daily 180-minutes conventional, occupational, and respiratory therapy (including limbs mobilization, muscle tone normalization, muscle strengthening, electrical muscle stimulation, and transfer, gait, and balance training), 6 days a week for 8 weeks. At T11, a brain MRI showed an enlargement and a numerical increment of the brainstem lesions found at T6 (Fig. 1B). At T12 (i.e., 2 months after admission), the patient was still unable to standing and ambulation, and complained also of a moderate cognitive deterioration (as per Mini-Mental State Examination test) and left side spasticity (as per Modified Ashworth Scale) (Table 1). No respiratory or cardiovascular clinical impairment was evident; blood chemistry was normal except for slight leukocytosis. Therefore, the patient was provided with additional 45-minutes of gait training by using LokomatPro (Hocoma AG; Volketswil, Switzerland), 45-minutes of upper limb training by using ArmeoSpring (Hocoma AG), and 30-minutes of cognitive rehabilitation by using the Virtual Reality Rehabilitation System Environment (VRRS) (Khymeia Group; Noventa Padovana, Italy). All these adjunctive treatments were carried daily, 6 days a week.

**Table 1:**
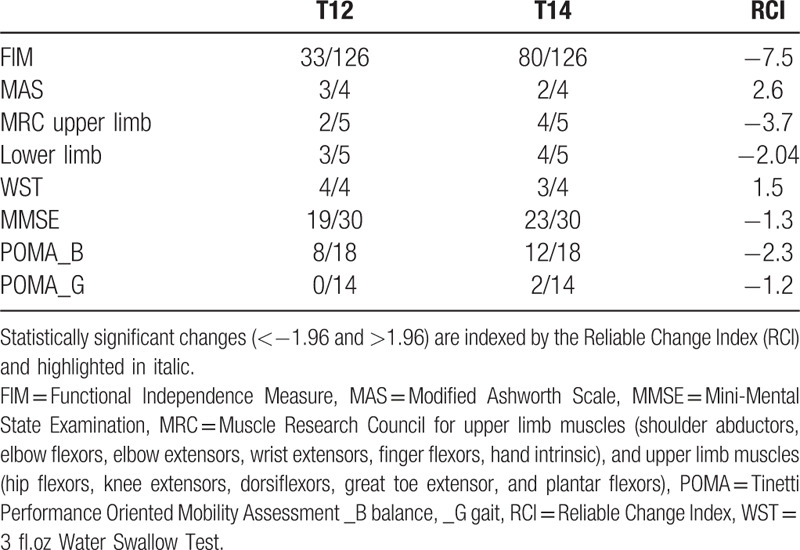
Clinical scores.

We adopted these robotic devices as the robotic limb orthoses combined with a body/limb weight support system (and a treadmill in the case of the LokomatPro) provide patients with intensive, repetitive, and task-oriented step/upper limb exercises that the patient can perform longer and with minor effort.^[11]^ Specifically, LokomatPro system guides patient's legs on the treadmill according to a preprogrammed physiological gait pattern, which is transmitted to both the treadmill movement and the motorized gait orthoses to induce physiological stance and swing phases. Both the body weight support and the specific level of guidance provided by the motorized gait orthoses were adapted to the patient's clinical condition to either achieve enhancements in gait speed, endurance, and gait quality and minimizing destructive compensatory gait pattern and avoidable stress to the patient.^[12]^

ArmeoSpring provides the patient with game-like functional limb movements and exercises within an interactive 2DVR environment (via computer screen). The exoskeleton is equipped with an upper limb weight support and allows movements related to reach and retrieval function, pronation, supination, wrist flexion and extension, and grasps and release. Thus, movements involving shoulder, elbow, wrist, and finger muscle and joints within a specified workspace are stimulated. The upper limb weight support provided by the limb orthosis was adapted to the patient's clinical condition.^[13]^

Further, employing virtual reality (VR) environment (i.e., VRRS) provides the patient with different kinds of motor tasks in which to hold a real manipulable object while interacting with a VR scenario.^[14]^ Specifically, we included a series of exercises involving attention, memory (verbal and visuospatial), spatial cognition, ocular-manual coordination, gnosis abilities, problem solving, executive function, and constructive praxis.^[13]^

The patient well tolerated the entire robot-aided training program, without any adverse event to be reported (including falls, muscle/tendon/joint strain, skin irritation). After 2 months (T14), the patient got a significant improvement in global functional disability, spasticity, cognitive impairment degree, and balance (Table 1). Significance of changes between T12 and T14 was assesses using the reliable change index. Consequently, the patient was discharged to continue the rehabilitation program in outpatient regimen.

## 3 Discussion

To the best of our knowledge, this is the first report on the application of a robotic-aided rehabilitative program in a patient with BRN. To date, there is no univocally effective treatment of cerebral radionecrosis.^[15]^ Conservative management mainly includes corticosteroid therapy,^[15]^ hyperbaric oxygen, anticoagulants, and monoclonal antibodies (e.g., bevacizumab); invasive procedures like craniotomy and lobectomy are used in drug-resistant conditions, but they are associated with a high risk of morbidity.^[5]^

Rehabilitation therapy is a cornerstone in the management of all brain tumor patients.^[7,16,17]^ Nonetheless, a rehabilitation plan should be always developed also in brain tumor complications and regardless of the rehabilitation setting.^[17]^ Rehabilitation setting and protocol have to be carefully tailored to patient tolerance, level of assistance in mobility, and activities of daily living, in keeping with the sensitivity of the case.^[17]^ Thus, an inadequate (in positive or negative) degree of conventional physiotherapy may result in a lack of benefit for the patient in terms of functional outcome.

Our case suggests that robot-aided rehabilitation can help to overcome the limitations to bear conventional physiotherapy due to patient's clinical condition. In fact, our patient showed a clear improvement in nearly all the outcomes when provided with the intensive robot-aided rehabilitative protocol, despite the corticosteroid treatment started since T6, the conventional physiotherapy since T10, and the worsening of MRI lesions from T6 to T11.

The strength point of robotic rehabilitation may depend on the fact that upper and lower extremity orthotic devices may work better in the patients who have persistent weakness and joint instability compared to those with a lesser extent of functional impairment.^[18,19]^ Further, robotic devices equipped with body weight support allow enhancing the effects of functional training by providing the patient with highly intensive, repetitive, precise, and task-oriented motor and cognitive tasks, in addition to conventional rehabilitation.^[10,20]^ Therefore, robot-aided intervention may be particularly indicated in patients with significant physical limitation, as in the case of BRN.

VR-based devices help in reducing the motor dysfunction in brain tumor patients, by emphasizing retraining and substitution of intact abilities and compensatory approaches.^[21]^ Thus, conjugating motor and cognitive rehabilitation may be of significant help in managing BRN-induced motor and cognitive limitations.

It is also hypothesizable that advanced neurorehabilitation may foster a higher entrainment of neuroplasticity mechanisms.^[22]^ It is known that neuroplasticity is the key mechanism by which the recovery processes following brain injury take place,^[23]^ and it is prompted by daily intensive neurorehabilitation.^[23]^ However, we can only speculate on the higher neuroplasticity effect of neurorobotics rehabilitation, as we did not perform neuroplasticity measures.

Notably, our intervention was safe for the patient. He indeed well tolerated the entire robot-aided training program, without any adverse event to be reported (including falls, muscle/tendon/joint strain, and skin irritation). The safety of robot-aided training program was mainly guaranteed by the use of joint sensors to monitor the force information of the subjects during the movement (thus avoiding too high reaction force caused by the muscle tension); a servomechanism preventing muscle strain when the patient appeared with muscle spasm, and controlling movement speed and the displacement of the robot; and a working space limited to a reasonable range of movements. Further, a trained operator set and controlled the parameters of the driving device and monitored the robot motion state in real time. Last, the patient was instructed and put in a position to push a press-and-stop button whether necessary.

One may concern that the improvement we observed could be attributed to the prolonged steroid therapy, spontaneous recovery, or both. However, 6 months of steroid therapy were ineffective, and steroid effectiveness usually occurs rapidly due to the resolution of the edema.^[24]^ Thus, spontaneous recovery can be ruled out, also because the MRI pattern was even worse at T11.

In conclusion, our case suggests the usefulness and safety of neurorobotics intensive rehabilitation in BRN to minimize the loss of neurological function, improve functional outcome, and reduce disability burden. Future studies should investigate whether an earlier, even multidisciplinary rehabilitative treatment could lead to better functional outcome in patients with BRN.

## Author contributions

**Conceptualization:** Francesco Tartamella.

**Data curation:** Maria Francesca Pisano, Adele Cacioppo, Simona Licari, Deborah Caradonna.

**Formal analysis:** Maria Francesca Pisano, Adele Cacioppo, Simona Licari, Deborah Caradonna.

**Funding acquisition:** Simona Portaro.

**Project administration:** Simona Portaro.

**Resources:** Simona Portaro.

**Supervision:** Rocco Salvatore Calabrò, Placido Bramanti, Antonino Naro.

**Validation:** Rocco Salvatore Calabrò, Placido Bramanti, Antonino Naro.

**Visualization:** Rocco Salvatore Calabrò, Placido Bramanti, Antonino Naro.

**Writing – original draft:** Francesco Tartamella, Antonino Chillura.

**Writing – review & editing:** Rocco Salvatore Calabrò, Antonino Naro.

## References

[1] Blanchard P Lee A Marguet S MAC-NPC Collaborative Group. Chemotherapy and radiotherapy in nasopharyngeal carcinoma: an update of the MAC-NPC meta-analysis. Lancet Oncol 2015;16:645–55. 25957714 10.1016/S1470-2045(15)70126-9

[2] Afrantou T Natsis KS Aristomenis A Brainstem radionecrosis in a patient with nasopharyngeal carcinoma. Can J Neurol Sci 2017;44:734–5. 28854994 10.1017/cjn.2017.225

[3] Hsu YC Wang LF Lee KW Cerebral radionecrosis in patients with nasopharyngeal carcinoma. Kaohsiung J Med Sci 2005;21:452–9. 16302448 10.1016/S1607-551X(09)70150-0 PMC11918143

[4] Lahlali F Echchikhi Y Chbili R Brain radionecrosis in patients irradiated for nasopharyngeal carcinoma: about four cases. Int Ann Med 2017 1.

[5] Li Y Shi X Rong X Neurosurgery and prognosis in patients with radiation-induced brain injury after nasopharyngeal carcinoma radiotherapy: a follow-up study. Radiat Oncol 2013;8:88. 23578338 10.1186/1748-717X-8-88 PMC3653741

[6] Kim HM Hong BY Lee JI Pontine necrosis related with radiation therapy, complicated with spontaneous hemorrhage. Brain Neurorehabil 2017;10:e1.

[7] Khan F Amatya B Ng L Multidisciplinary rehabilitation after primary brain tumor treatment. Cochrane Database Syst Rev 2013;1:CD009509. 10.1002/14651858.CD009509.pub2 23440839

[8] Wong ST Loo KT Yam KY Results of excision of cerebral radionecrosis: experience in patients treated with radiation therapy for nasopharyngeal carcinoma. J Neurosurg 2010;113:293–300. 20151776 10.3171/2010.1.JNS091039

[9] Calabrò RS Cacciola A Bertè F Robotic gait rehabilitation and substitution devices in neurological disorders: where are we now? Neurol Sci 2016;37:503–14. 26781943 10.1007/s10072-016-2474-4

[10] Colombo R Sanguineti V Rehabilitation Robotics: Technology and Applications. London: Elsevier Academic Press; 2018.

[11] Krebs HI Volpe BT Rehabilitation robotics. Handb Clin Neurol 2013;110:283–94. 23312648 10.1016/B978-0-444-52901-5.00023-X PMC4688009

[12] Chung BP Effectiveness of robotic-assisted gait training in stroke rehabilitation: a retrospective matched control study. Hong Kong Physiother J 2016;36:10–6. 30931034 10.1016/j.hkpj.2016.09.001 PMC6385094

[13] De Luca R Russo M Naro A Effects of virtual reality-based training with BTs-Nirvana on functional recovery in stroke patients: preliminary considerations. Int J Neurosci 2018;128:791–6. 29148855 10.1080/00207454.2017.1403915

[14] Tieri G Morone G Paolucci S Iosa M Virtual reality in cognitive and motor rehabilitation: facts, fiction and fallacies. Expert Rev Med Devices 2018;15:107–17. 29313388 10.1080/17434440.2018.1425613

[15] Shaw PJ Bates D Conservative treatment of delayed cerebral radiation necrosis. J Neurol Neurosurg Psychiatry 1984;47:1338–41. 6512555 10.1136/jnnp.47.12.1338 PMC1028144

[16] Geler-Kulcu D Gulsen G Buyukbaba E Ozkan D Functional recovery of patients with brain tumor or acute stroke after rehabilitation: a comparative study. J Clin Neurosci 2009;16:74–8. 19022673 10.1016/j.jocn.2008.04.014

[17] Vargo M Brain tumor rehabilitation. Am J Phys Med Rehabil 2011;90: 5 Suppl 1: S50–62. 10.1097/PHM.0b013e31820be31f 21765264

[18] Hussain A Budhota A Contu S Quantitative assessment of motor functions post-stroke: responsiveness of upper-extremity robotic measures and its task dependence. IEEE Int Conf Rehabil Robot 2017;2017:1037–42. 28813958 10.1109/ICORR.2017.8009386

[19] Wei XJ Tong KY Hu XL The responsiveness and correlation between Fugl-Meyer Assessment, Motor Status Scale, and the Action Research Arm Test in chronic stroke with upper-extremity rehabilitation robotic training. Int J Rehabil Res 2011;34:349–56. 22044987 10.1097/MRR.0b013e32834d330a

[20] Gassert R Dietz V Rehabilitation robots for the treatment of sensorimotor deficits: a neurophysiological perspective. J Neuroeng Rehabil 2018;15:46. 29866106 10.1186/s12984-018-0383-x PMC5987585

[21] Yoon J Chun MH Lee SJ Kim BR Effect of virtual reality-based rehabilitation on upper-extremity function in patients with brain tumor: controlled trial. Am J Phys Med Rehabil 2015;94:449–59. 25251249 10.1097/PHM.0000000000000192

[22] Turner DL Ramos-Murguialday A Birbaumer N Neurophysiology of robot-mediated training and therapy: a perspective for future use in clinical populations. Front Neurol 2013;4:184. 24312073 10.3389/fneur.2013.00184 PMC3826107

[23] Dimyan MA Cohen LG Neuroplasticity in the context of motor rehabilitation after stroke. Nat Rev Neurol 2011;7:76–85. 21243015 10.1038/nrneurol.2010.200 PMC4886719

[24] Vellayappan B Tan CL Yong C Diagnosis and management of radiation necrosis in patients with brain metastases. Front Oncol 2018;8:395. 30324090 10.3389/fonc.2018.00395 PMC6172328

